# The Microscope in Daily Practice, (First Paper)

**Published:** 1875-09

**Authors:** I. N. Danforth

**Affiliations:** Lecturer on Pathology in Rush Medical College, Chicago


### THE MICROSCOPE IN DAILY PRACTICE.

(FIRST PAPER.)

By I. N. DANFORTH, M.D.,

Lecturer on Pathology in Rush Medical College, Chicago.

The great problem in connection with the microscope now-a-days is, *not* how to make it more "perfect," more complicated and more costly, but how to make it more available. Our students generally look upon the microscope as a kind of "luxury," only to be indulged in after the inevitable amputating case, or the more formidable "omnibus" case, has been purchased, exhibited and adored. And yet, every physician of experience would tell the student that the little pastime of cutting off arms and legs is generally far to seek, while the microscope is constantly and practically useful. Scarcely a day goes by, that a physician in active practice could not, with great benefit, employ the microscope for purposes of diagnosis; in a vast number of cases it is essential, and in not a few it simply comes to this—no microscope, no *certain* diagnosis. A physician has no right to go blundering along through the bogs and fens of guess-work, when for a moderate outlay he can command the means necessary to reach *certainty*.

More than this, the microscope is a constant educator; it widens the boundaries of the student's knowledge; it turns his thoughts into new channels; it reveals the secrets of nature's dainty handiwork; it shows him forms of beauty that were else unseen; it tempts him to a wider range of scientific study; and by so much as it adds to his culture, and enlarges the range of his mental vision, does it increase his power and influence in the community. Nor is it intended to limit the term "student" to the young man just from the lecture room. I can point to a man of wealth and broad scientific culture, well up among the "sixties," gray-haired and spectacled, who has lately become the possessor of a fine microscope, and I have never seen a more delighted, enthusiastic or pains-taking "student" of microscopy.

Considering, then, the vast and growing importance of the microscope in practical medicine, and as a means of scientific research, I do not think I shall need to apologize to the readers of the JOURNAL AND EXAMINER, if I print therein a few brief articles concerning the instrument and its proper and profitable employment.

As a general rule, the first thing in order is the purchase of a microscope; and it is also quite true to say that as a general rule the purchaser gets cheated. The uninstructed purchaser asks at once of a given microscope, "how many diameters does it magnify?" whereas "diameters" are about the cheapest, as well as the most useless, quality in connection with a microscope. Not one in ten amateurs takes pains to inform himself in regard to the points really desirable in a good microscope; hence he is more than likely to buy the instrument that furnishes the greatest array of polished brass, the greatest number of milled-headed screws, and the most formidable tangle of complicated tackle. At all events, he is compelled to take the judgment of dealers, and dealers look at microscopes from the stand-point of profit and loss. A student once told me that his preceptor possessed a microscope which magnified *five thousand diameters* and cost *fifty dollars!* I have envied that "preceptor" ever since. If I could have my say, I would advise every one who contemplates buying a microscope, to first procure some standard work on practical microscopy, and carefully read it; it would save a multitude of blunders. The question immediately comes up, "what work is the best?" I believe the best physicians' hand-book of microscopy yet published, is that of Richardson, of Philadelphia; it is practical, concise, cheap, and, in the main, accurate and reliable. Among more elaborate and costly works, Beale's "How to Work with the Microscope," is perhaps the best; Frey's "Microscope and Microscopic Technology," next; and Carpenter's "Microscope and its Revelations," last. This, however, is a matter of opinion, and I am simply giving *my* opinion.

I am very positive, however, that the beginner can very profitably study either of these excellent authorities before buying a microscope.

What constitutes a good microscope?

1st. It should stand firmly upon a broad and somewhat heavy tripod base, and the three arms of the tripod should be sufficiently long to insure perfect steadiness, in whatever position the instrument is placed.

2d. It should be perfectly free from lateral movement or oscillation, when the focus is changed by turning either of the adjustment screws, and this should be equally true whether the instrument be vertical, inclined or horizontal.

3d. The body or tube ought always to be attached to the base by a firm joint, so that any position from vertical to horizontal can be secured. This is an exceedingly important point. Every practical microscopist knows how very tiresome it is to work with an instrument which is fixed in the vertical position like the old-fashioned Oberhauser instruments, and some of those now made in Germany.

4th. The stage should be large enough not only for the free movement of objects fixed upon glass slides, but for the attachment of stage forceps, a frog plate and an animalcule cage; a revolving diaphragm, having five or six openings, should always be fixed below the stage; the mirror should be large, should have two surfaces, plane and parabolic, and should be capable of free lateral movement, so as to enable the observer to employ oblique light when desirable. No stand is worth much which does not combine these indispensable requisites. So much for the "stand," or mechanical portion.

The optical portion of a good, serviceable microscope should include at least:

(*a*). A No. 1 eye piece—perhaps more generally called in this country the "A" eye piece. It is very common to have two eye pieces, Nos. 1 and 2, or the "A" and "B" eye piece, the latter possessing a higher amplifying power than the former; but I am persuaded that very little is gained by high eye pieces. The eye piece can only magnify the magnified image, and this can only be accomplished at the expense of definition and clearness. I suppose that the diatomists find great benefit from the use of high eye pieces, but for histological purposes they are not generally useful. It is far better to put the magnifying power into the objective.

(*b*). Two first-class objectives should accompany every physician's microscope, namely, an inch and a quarter inch, or a fifth. (By "inch" and "quarter inch" it is merely intended to say that the former has a focal distance of one inch, and the latter a quarter of that focal distance—statements that are perhaps never *exactly* but only *approximately* true.)

By "first-class" objectives, I do not, by any means, refer to those which are the most costly. The cost of an objective depends largely upon its "angle of aperture," and the "angle of aperture" is the "angle made by two lines from opposite sides of the aperture of the object glass with the point of focus of the lens."* Now the objective makers, being incited thereto by those enthusiasts who have gone crazy on the subject of "diatomes," have themselves gone crazy on the subject of "high angles," which, as Beale remarks, are "valuable for investigations upon many very delicate and thin structures, such as the diatomaceæ; but such powers are not well adapted for ordinary work,"—that is, for histological work. Some of the "high angle" quarters and fifths, of the best makers, cost as high as forty or fifty dollars, which is simply an absurd outlay of money, unless the purchaser proposes to spend his time in the comparatively useless work of "resolving" the markings on the more difficult diatomes, or other "test" objects. I believe the most useful quarter or fifth inch objectives for histological investigation and diagnostic purposes—indeed for the general wants of the practitioner—are those whose angular aperture is not over 70 or 80 degrees, costing, in

* Beale: "How to Work with the Microscope," 4th ed., p. 7.

currency, not more than from fifteen to twenty dollars, or at most, twenty-five dollars. The inch objectives do not vary so much. There is little difficulty in procuring a good one; those made by Tolles and Wales are especially good; and I may add, that Hartnack's are of equal excellence.

(*c*). A condensing lens, or "bull's eye," should accompany every physician's microscope. It is all but as indispensable as an objective. It is constantly needed for the examination of objects by "reflected" light, and at the same time it furnishes, without extra cost, a large "hand magnifier," which is exceedingly useful for very many purposes.

In this brief list, I have included everything *necessary* for a really good and serviceable physician's microscope; and such a microscope every physician ought to possess and *use*, just as constantly as he does his stethoscope, his fever thermometer, or his urinometer.

In a future paper, I will endeavor to show the minimum cost of such an instrument, and also say something as to the merits of the instruments of different makers.

---

Glycerin in the Treatment of Diabetes.—M. Garnier has addressed a note to the Académie des Sciences on the employment of glycerin in the treatment of glycosuria. M. Schutzen, of Dorpat, has shown by his researches that glycerin either alone or associated with tartaric acid, and taken in the dose of half an ounce to an ounce and a half in the course of twenty-four hours, constitutes a valuable adjuvant to the régime especially adapted to glycosuria. M. G. has himself made use of purified glycerin, and has made it more palatable by mixing it with a certain amount of alcohol and of aromatic substances (mint, anise, bitter orange.) The employment of glycerin has aided him greatly as well as other patients.—*Bull. Gén. de Thérap.*, No. 10, 1875.

